# Preoperative evaluation of mediastinal lymph nodes in non-small cell lung cancer using [^68^Ga]FAPI-46 PET/CT: a prospective pilot study

**DOI:** 10.1007/s00259-024-06669-y

**Published:** 2024-03-07

**Authors:** Yeon-koo Kang, Kwon Joong Na, Jimyung Park, Nakwon Kwak, Yun-Sang Lee, Hongyoon Choi, Young Tae Kim

**Affiliations:** 1 Department of Nuclear Medicine, Seoul National University Hospital, 101 Daehak-ro, Jongno-gu, Seoul, 03080 Republic of Korea; 2 Department of Thoracic and Cardiovascular Surgery, Seoul National University Hospital, 101 Daehak-ro, Jongno-gu, Seoul, 03080 Republic of Korea; 3 Division of Pulmonary and Critical Care Medicine, Department of Internal Medicine, Seoul National University Hospital, Seoul National University College of Medicine, Seoul, Republic of Korea; 4 Department of Nuclear Medicine, Seoul National University College of Medicine, Seoul, Republic of Korea; 5 Cancer Research Institute, Seoul National University College of Medicine, Seoul, Republic of Korea; 6 Institute of Radiation Medicine, Seoul National University Medical Research Center, Seoul, Republic of Korea

**Keywords:** Lung cancer, Mediastinal lymph node, Fibroblast activation protein, [^68^Ga]FAPI-46, PET/CT

## Abstract

**Purpose:**

Mediastinal nodal staging is crucial for surgical candidate selection in non-small cell lung cancer (NSCLC), but conventional imaging has limitations often necessitating invasive staging. We investigated the additive clinical value of fibroblast activation protein inhibitor (FAPI) PET/CT, an imaging technique targeting fibroblast activation protein, for mediastinal nodal staging of NSCLC.

**Methods:**

In this prospective pilot study, we enrolled patients scheduled for surgical resection of NSCLC based on specific criteria designed to align with indications for invasive staging procedures. Patients were included when meeting at least one of the following: (1) presence of FDG-positive N2 lymph nodes, (2) clinical N1 stage, (3) central tumor location or tumor diameter of ≥ 3 cm, and (4) adenocarcinoma exhibiting high FDG uptake. [^68^Ga]FAPI-46 PET/CT was performed before surgery after a staging workup including [^18^F]FDG PET/CT. The diagnostic accuracy of [^68^Ga]FAPI-46 PET/CT for “N2” nodes was assessed through per-patient visual assessment and per-station quantitative analysis using histopathologic results as reference standards.

**Results:**

Twenty-three patients with 75 nodal stations were analyzed. Histopathologic examination confirmed that nine patients (39.1%) were N2-positive. In per-patient assessment, [^68^Ga]FAPI-46 PET/CT successfully identified metastasis in eight patients (sensitivity 0.89 (0.52–1.00)), upstaging three patients compared to [^18^F]FDG PET/CT. [^18^F]FDG PET/CT detected FDG-avid nodes in six (42.8%) of 14 N2-negative patients. Among them, five were considered non-metastatic based on calcification and distribution pattern, and one was considered metastatic. In contrast, [^68^Ga]FAPI-46 PET/CT correctly identified all non-metastatic patients solely based on tracer avidity. In per-station analysis, [^68^Ga]FAPI-46 PET/CT discriminated metastasis more effectively compared to [^18^F]FDG PET/CT-based (AUC of ROC curve 0.96 (0.88–0.99) vs. 0.68 (0.56–0.78), *P* < 0.001).

**Conclusion:**

[^68^Ga]FAPI-46 PET/CT holds promise as an imaging tool for preoperative mediastinal nodal staging in NSCLC, with improved sensitivity and the potential to reduce false-positive results, optimizing the need for invasive staging procedures.

**Supplementary information:**

The online version contains supplementary material available at 10.1007/s00259-024-06669-y.

## Introduction

In non-small cell lung cancer (NSCLC), accurate mediastinal nodal staging is crucial for selecting surgery candidates [1, 2] and predicting outcomes [3]. Currently, contrast-enhanced CT (CECT) and 2-deoxy-2-[^18^F]fluoro-D-glucose ([^18^F]FDG) PET/CT are the imaging standards for preoperative staging [1]. Although FDG PET/CT offers greater sensitivity and specificity [4–6], it has intrinsic limitations in distinguishing between inflammation and metastasis, resulting in suboptimal accuracy particularly in tuberculosis-endemic regions [7–9]. In a study from a tuberculosis-endemic country, [^18^F]FDG PET/CT demonstrated a sensitivity of 81% and specificity of 73% in diagnosing metastatic mediastinal lymph nodes based solely on their FDG avidity. However, specificity increased to 89%, while sensitivity decreased to 75%, after considering calcification and distribution patterns [8]. This trade-off between sensitivity and specificity was reproduced in other studies [7, 9]. These limitations have led to needs for invasive procedures, such as endobronchial ultrasound-guided transbronchial needle aspiration (EBUS-TBNA) [2, 10–12].

EBUS-TBNA is a standard procedure for the preoperative mediastinal node evaluation, enabling pathologic sample acquisition and accurate staging [10, 11, 13, 14]. It has undeniably become the gold standard for identifying metastatic nodes in patients with high-risk factors or clinically suspected lesions, where accurately confirming N stages before proceeding to surgery is essential. However, EBUS-TBNA remains an invasive method, associated with patient discomfort, often requiring sedation, and carrying potential complications like bleeding, pneumothorax, and infection, although fatal outcomes are rare [15–17]. This necessitates refined strategy to select patients who truly require this invasive procedure. Additionally, given that EBUS-TBNA cannot assess certain lymph nodes, specifically stations 5, 6, and 9, the role of non-invasive imaging becomes crucial for accurate N-staging. Consequently, an additional non-invasive modality that can accurately detect nodal metastases is needed as a tool to optimize the indications for EBUS-TBNA.

Recently, fibroblast activation protein inhibitor (FAPI) PET/CT, which targets fibroblast activation protein (FAP) overexpressed in the solid tumor microenvironment, has emerged as a promising tool in oncologic imaging [18–20]. This technique has shown increased tracer uptake in various malignancies, providing favorable diagnostic yields, even where [^18^F]FDG PET/CT faces limitations [21–23]. Previous investigations into NSCLC have shown its comparable efficacy in identifying primary tumors and superior sensitivity for metastatic lesions [24–26]. Although these studies provide valuable insights into the diagnostic capabilities of FAPI PET/CT in lung cancer, their assessment of its utility focused on preoperative patients is partly limited due to the lack of postoperative pathologic results and the inclusion of patients with advanced or recurrent disease. As evidence supporting the performance of FAPI PET/CT grows across various cancer types, it becomes crucial to prove its role in specified clinical contexts [27].

In this context, this prospective pilot study focused on the potential of [^68^Ga]FAPI-46 PET/CT for preoperative mediastinal nodal staging in NSCLC, specifically targeting patients who may require invasive nodal staging due to suspicions of locally advanced disease from conventional imaging. Our goal was to evaluate the additive value of [^68^Ga]FAPI-46 PET/CT in nodal staging compared to conventional imaging, especially in identifying N2 stage, a pivotal aspect in making management plan decisions for surgical candidates.

## Methods

### Patients and study design

This is a single-center prospective pilot study. NSCLC patients planned for curative surgery were potential candidates. Patients underwent conventional imaging studies, including [^18^F]FDG PET/CT, before recruitment. Since the focus was the role of FAPI PET/CT in situations requiring invasive nodal staging, inclusion criteria were aligned with potential EBUS-TBNA indications based on widely accepted clinical practice guidelines [2, 28]. Patients were eligible when meeting at least one of the following: (1) FDG-positive N2 nodes, (2) clinical N1 stage in non-invasive staging, (3) central lung cancer or ≥3 cm diameter, and (4) adenocarcinoma with high FDG uptake. Patients scheduled for any therapies prior to surgery, including chemotherapy and radiation therapy, were excluded from the study. Additional exclusion criteria encompassed the presence of other malignancies and intolerance to PET scanning.

Recruited patients underwent [^68^Ga]FAPI-46 PET/CT and received curative surgery by two board-certified surgeons (Y.T.K. and K.J.N.) within 2 days. All patients underwent lung cancer surgery. Anatomic segmentectomy, lobectomy, pneumonectomy, or wedge resection was performed based on the patient’s clinical stage and the surgeon’s preoperative plan. Systematic mediastinal lymph node dissection (MLND) that covers ipsilateral hilar and ipsilateral mediastinal lymph nodes was planned for all patients, but mediastinal lymph node sampling was performed in one patient with heavy lymph node anthracosis. The decision regarding which stations to resect for each patient was made based on intraoperative assessments by the surgeons as in routine clinical practice, focusing on the presence and significance of grossly identifiable nodal involvement. Histopathologic analyses were performed in all primary tumors and resected lymph nodes.

The diagnostic value of [^68^Ga]FAPI-46 PET/CT in diagnosing N2 mediastinal nodal metastasis was assessed using postoperative pathologic results as reference standards and compared to [^18^F]FDG PET/CT. In our study, we included only lymph node stations classified as N2 for each patient, based on the following considerations. First, N2 metastasis plays a major pivotal role in deciding on curative surgery. Second, N3 nodes are typically not resected in routine surgical procedures, and hilar, peribronchial, or intrapulmonary nodes present challenges in correlating postoperative pathologic results with cross-sectional images, thereby complicating the accurate evaluation of diagnostic accuracy.

**Fig. 1:**
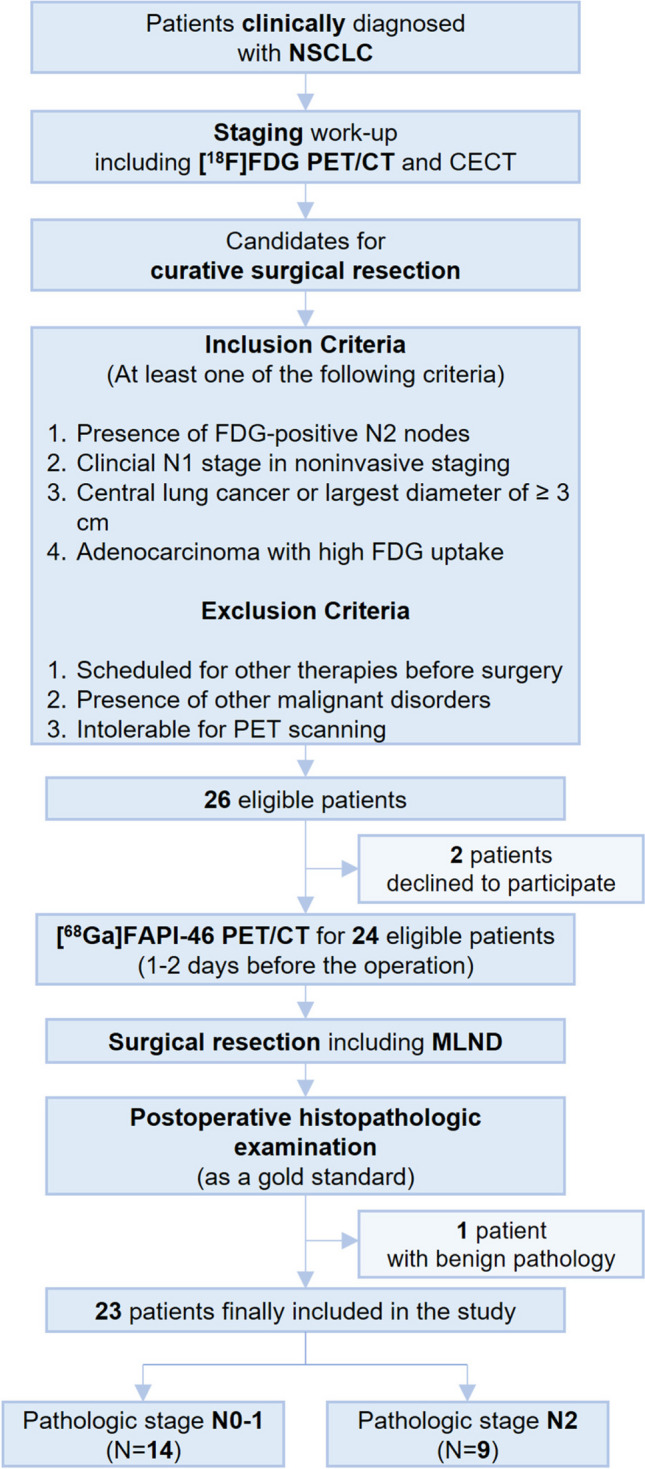
Overall study flow. *FAPI* fibroblast activation protein inhibitor; *CECT* contrast-enhanced computed tomography; *MLND* mediastinal lymph node dissection

The study design is summarized in Figure 1. This study was approved by the institutional review board (No. H-2112-110-1284), and written informed consent was acquired from all patients.

### [^18^F]FDG and [^68^Ga]FAPI-46 PET/CT imaging

[^18^F]FDG PET/CT scans were performed in line with routine clinical protocols. Patients received an intravenous injection of 0.14 MBq/kg [^18^F]FDG, followed by non-enhanced CT and PET scans (2 min/bed) at post-injection 60 min, covering from the skull vertex to the proximal thigh. Similarly, for [^68^Ga]FAPI-46 PET/CT, non-enhanced CT and PET scans (2 min/bed) were conducted 60 min after the intravenous injection of 185 MBq of [^68^Ga]FAPI-46. Both PET/CT scans were conducted using dedicated PET/CT scanners (Biograph mCT 40 or 64, Siemens Healthineers, Erlangen, Germany). The acquired PET images of both scans were reconstructed via ordered subset expectation maximization algorithms (2 iterations, 21 subsets), with time of flight and point spread function. CT-based attenuation correction and post-reconstruction Gaussian filter (FWHM 5 mm) were applied, and the matrix size was set to 200 × 200. Integrated PET/CT images were analyzed using dedicated software (Syngo.via, Siemens Healthineers, Erlangen, Germany).

### Per-patient assessment

The accuracy of [^68^Ga]FAPI-46 PET/CT and [^18^F]FDG PET/CT in diagnosing N2 metastasis was assessed through two approaches, per-patient visual assessment and per-station quantitative analysis, using pathologic results as the reference standard. In per-patient assessment, two nuclear medicine physicians (Y.K. and H.C.) with over 9 years of PET reading experience reviewed scans to determine the presence of nodal metastasis based on each scan. In [^68^Ga]FAPI-46 PET/CT, lymph nodes exhibiting tracer uptake more than twice the intensity of the mediastinal blood pool on visual assessment were considered metastatic. For [^18^F]FDG PET/CT, presumed metastatic nodes needed to meet the following additionally: non-symmetric lesion distribution and the absence of gross calcification, following routine clinical practices and previous studies [7, 8]. The per-patient positivity for N2 metastasis was determined separately for each PET/CT scan.

### Per-station assessment

For the per-station assessment, each patient’s individual nodal stations were quantitatively evaluated. The nodal stations were defined according to the International Association for the Study of Lung Cancer lymph node map [29], and we specifically focused on “N2” stations: right upper paratracheal (2R), right lower paratracheal (4R), subcarinal (7), right paraesophageal (8R), and right pulmonary ligament (9R) stations for right lung cancer, and left lower paratracheal (4L), subaortic (5), paraaortic (6), subcarinal (7), left paraesophageal (8L), and left pulmonary ligament (9L) stations for left lung cancer. All resected nodal stations were included in the analysis regardless of FDG or FAPI avidity. For quantitative analyses, volumes of interest (VOIs) were drawn on the hottest lesion from each nodal station, and tumor-to-background ratio (TBR) to represent tracer uptake was calculated as the maximum standardized uptake value (SUV_max_) of the lesion divided by mean mediastinal blood pool activity. Blood pool activity was measured in a spherical VOI with a 1.5 cm diameter placed in the ascending aorta, excluding aortic wall activity. TBR of the stations in both [^68^Ga]FAPI-46 and [^18^F]FDG PET/CT scans were tested for distinguishing metastasis, respectively.

### Statistical analysis

Demographic data were presented using subject numbers and proportions for categorical parameters and averages and standard deviations for continuous parameters. The per-patient analysis evaluated the diagnostic performance of PET/CT scans in detecting metastasis using sensitivity and specificity. In the per-station analysis, the quantitative parameters of the nodal stations were compared between pathologically proven benign and metastatic nodes using Mann–Whitney test. The parameters were assessed for their diagnostic performance to detect metastasis using receiver operating curve (ROC) analysis. ROC curves from different diagnostic modalities were compared using DeLong’s method [30]. Optimal cutoff values were determined using Youden’s index, and sensitivity and specificity using these values were presented. Statistical analyses were performed using MATLAB R2023b (MathWorks, Natick, MA) and the open-source library SciPy 1.7.3 (https://github.com/scipy/scipy).

## Results

### Patients

Twenty-six candidates were initially identified based on the inclusion criteria from May 2022 to July 2023. Among them, two patients declined to participate, and one patient was excluded due to a benign pathology of the primary lesion confirmed by final postoperative pathologic examination. As a result, a total of 23 patients (64.4 ± 7.1 years, M:F = 16:7) were finally enrolled. Most patients underwent lobectomy, while two patients received pneumonectomy, and one patient each underwent wedge resection, segmentectomy, and bilobectomy. Postoperative histopathologic analysis confirmed the presence of NSCLC in all participants, with the majority being diagnosed with adenocarcinoma (18 patients). Pathologic T stage mainly ranged from T1 to T3, except two patients. The mean size of primary tumor on CT was 3.6 ± 1.9 cm, which was deemed suitable for the quantitative evaluation on PET images. Primary tumor SUV_max_ measured on [^68^Ga]FAPI-46 PET/CT and [^18^F]FDG PET/CT was 8.4 ± 4.6 and 10.4 ± 6.5, respectively, without significant inter-scan difference (*P* = 0.076). Demographic factors and clinical characteristics of the patients are provided in Table 1.

**Table 1:** Clinical characteristics of patients

Factor	Mean ± SD or *N* (%)
Age (y)	66.0 ± 8.2
Gender (M:F)	16:7
Smoking history	15 (65.2%)
Primary tumor size on CT (cm)	3.6 ± 1.9
Histology	
Adenocarcinoma	18 (78.3%)
Non-adenocarcinoma^*^	5 (11.7%)
Resection	
Wedge resection	1 (4.3%)
Segmentectomy	1 (4.3%)
Lobectomy	18 (78.3%)
Bilobectomy	1 (4.3%)
Pneumonectomy	2 (8.7%)
Pathologic T stage	
T1	8 (34.8%)
T2	8 (34.8%)
T3	5 (21.7%)
T4	2 (8.7%)
Pathologic N stage	
N0	12 (52.2%)
N1	2 (8.7%)
N2	9 (39.1%)
Lobe of primary tumor	
Right upper	9 (39.1%)
Right middle	1 (4.3%)
Right lower	6 (26.1%)
Left upper	3 (13.0%)
Left lower	4 (17.4%)
Primary tumor SUV_max_ (FAPI PET/CT)	8.4 ± 4.6
Primary tumor SUV_max_ (FDG PET/CT)	10.4 ± 6.5
Interval between FDG PET/CT and FAPI PET/CT (days)	49 ± 34

^*^Including squamous cell carcinoma, adenosquamous carcinoma, large cell carcinoma, and sarcomatoid carcinoma

*FAPI*, fibroblast activation protein inhibitor; SUV_max_, maximum standardized uptake value

### Per-patient assessment

The presence of metastasis in N2 stations was detected in nine patients (39.1%) through histopathologic analysis. Based on visual assessment, [^68^Ga]FAPI-46 PET/CT accurately identified eight of patients with N2 metastasis and correctly ruled out metastasis in all patients without pathologic N2 involvement (Table 2). In contrast, [^18^F]FDG PET/CT accurately detected five N2-positive patients and misdiagnosed one N2-negative patient to be positive. The per-patient sensitivity and specificity were 0.89 (95% CI 0.52–1.00) and 1.00 (0.77–1.00) for [^68^Ga]FAPI-46 PET/CT and 0.56 (0.21–0.86) and 0.93 (0.66–1.00) for [^18^F]FDG PET/CT, respectively.

**Table 2:** Per-patient and per-station diagnosis of N2 metastasis

Per-patient analysis (visual assessment)					
	FAPI-positive	FAPI-negative	FDG-positive	FDG-negative	All
N2-positive	8 (88.9%)	1 (11.1%)	5 (55.6%)	4 (44.4%)	9
N2-negative	0 (0.0%)	14 (100.0%)	1 (7.1%)	13 (92.9%)	14
All	8	15	6	17	23
Per-station analysis (with determined optimal cutoff for quantitative parameters)					
	FAPI-positive	FAPI-negative	FDG-positive	FDG-negative	All
N2-positive	12 (92.3%)	1 (7.7%)	5 (38.5%)	8 (61.5%)	13
N2-negative	6 (9.7%)	56 (90.3%)	5 (8.1%)	57 (91.9%)	62
All	18	57	20	55	75

*FAPI*, fibroblast activation protein inhibitor

**Fig. 2:**
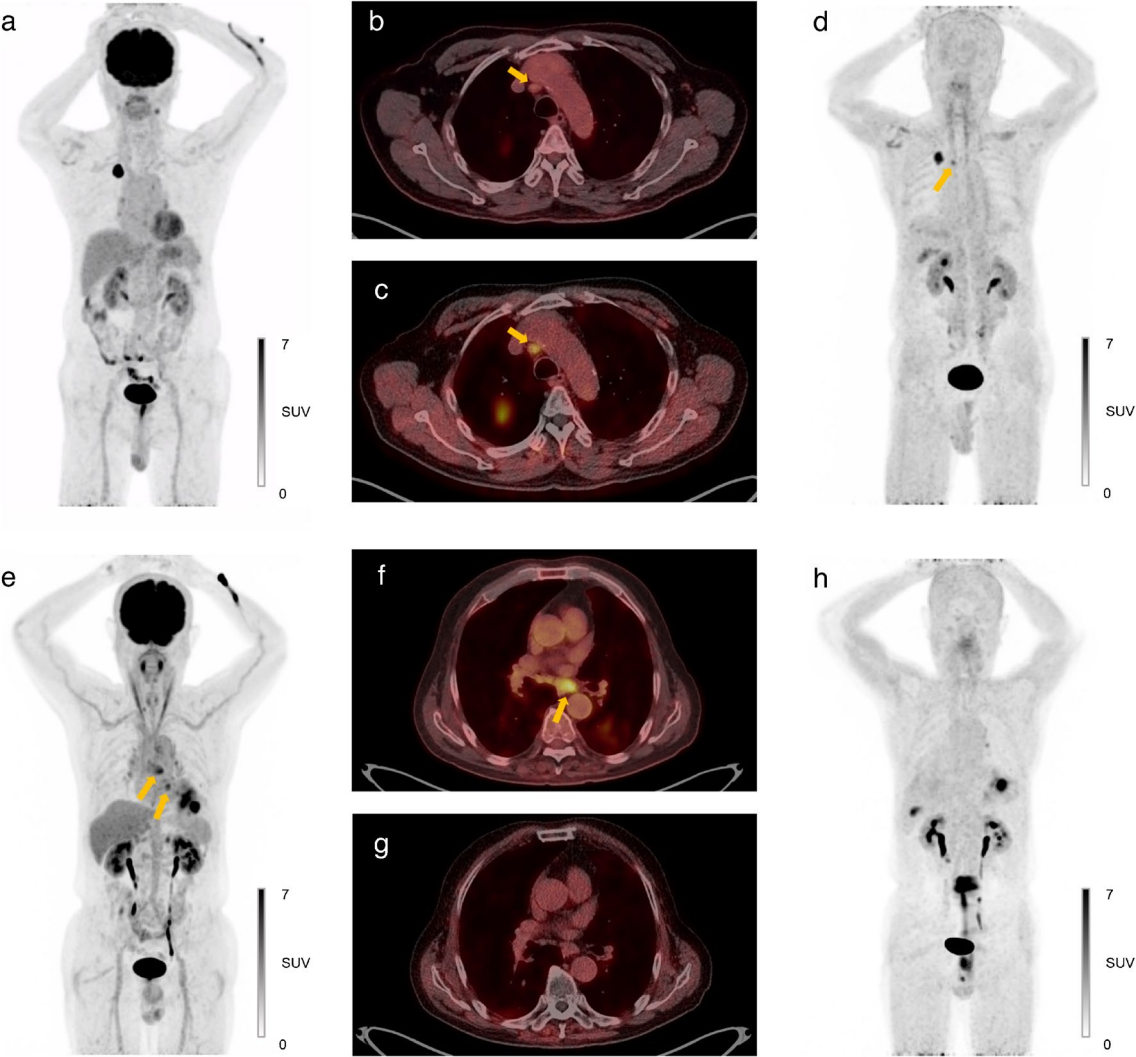
Representative N2-positive and N2-negative cases. **a**-**d** A 71-year-old male patient diagnosed with lung adenocarcinoma. Preoperative [^18^F]FDG PET/CT (**a**-**b**) did not exhibit any abnormal lesions except the primary tumor in the right upper lobe, whereas [^68^Ga]FAPI-46 PET/CT (**c**-**d**) revealed a lymph node with significant uptake in the right paratracheal area (*arrows*). The patient underwent lobectomy and MLND, and the paratracheal lymph node was confirmed to be metastatic. **e**-**h** A 79-year-old male patient diagnosed with squamous cell carcinoma of the left lower lobe. In preoperative [^18^F]FDG PET/CT (**e**–**f**), lymph nodes with increased uptake were detected in stations 8 and 9L (*arrows*). In contrast, the nodes did not exhibit significant tracer uptake on [^68^Ga]FAPI-46 PET/CT (**g**–**h**). EBUS-TBNA was performed before surgery, and the evidence of metastasis was not found on station 8. The patient underwent left lower lobectomy and MLND, and histopathologic analysis confirmed the absence of nodal metastasis. FAPI, fibroblast activation protein inhibitor; MLND, mediastinal lymph node dissection; EBUS-TBNA, endobronchial ultrasound-guided transbronchial needle aspiration

[^68^Ga]FAPI-46 PET/CT upstaged three patients (13.0% of subjects) from N1 to N2 compared to [^18^F]FDG PET/CT, who were all pathologically confirmed as N2 stage. Two patients were upstaged due to metastasis in station 2R (Figure 2a–d), and the other had metastases in stations 7 and 8.

**Fig. 3:**
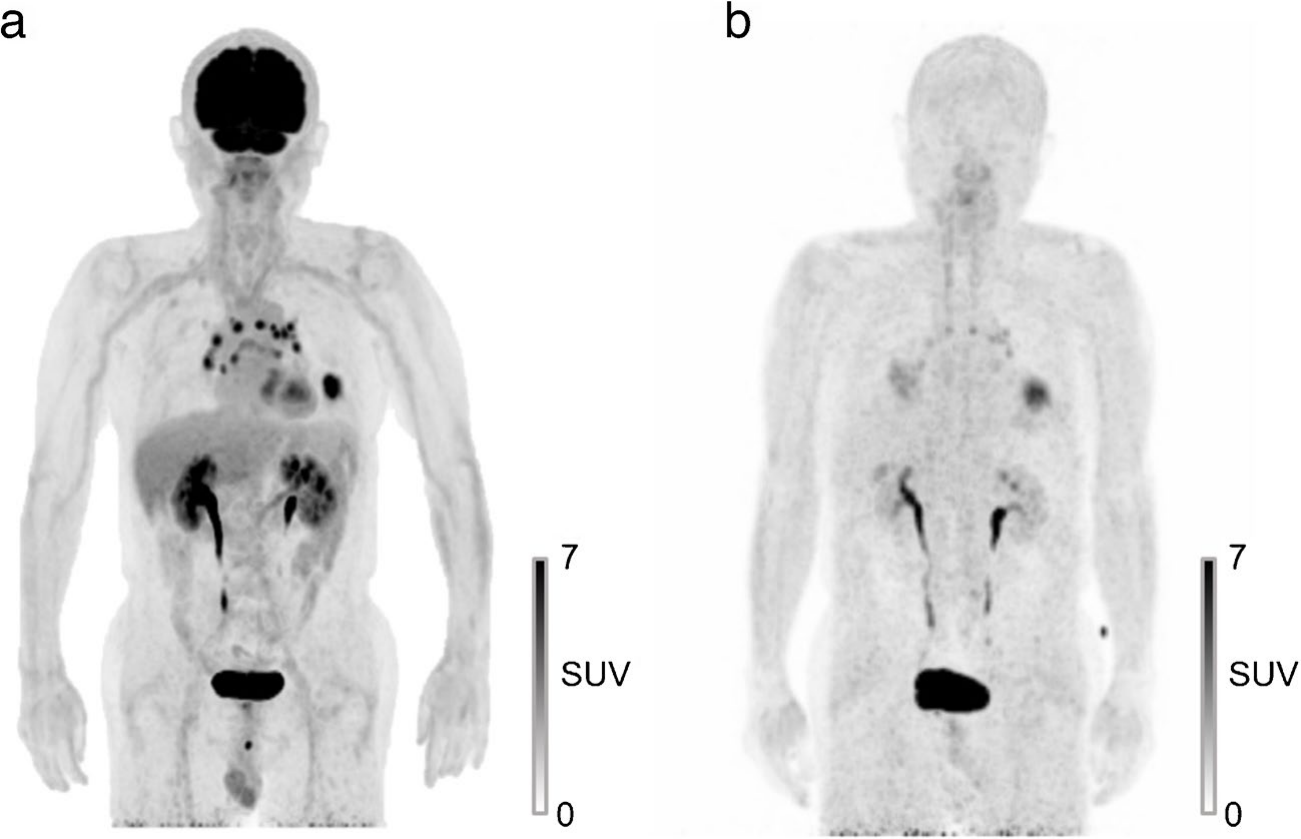
A case with multiple FDG-avid N2 nodes that were pathologically proven to be non-metastatic. A 75-year-old male patient diagnosed with lung adenosquamous carcinoma. In preoperative [^18^F]FDG PET/CT (**a**), multiple FDG-avid mediastinal lymph nodes were detected with the primary tumor in the left lower lobe, some of which showed increased size on CT. In contrast, the nodes exhibited only mild tracer uptake in [^68^Ga]FAPI-46 PET/CT (**b**). EBUS-TBNA was performed to exclude metastasis prior to surgery, and none of lesions was identified to be metastatic. The patient underwent lobectomy and MLND, and histopathologic analysis confirmed the absence of nodal metastasis

Among the fourteen N2-negative patients, six (42.9%) exhibited FDG-avid N2 lesions. One patient was assessed to be positive (Figure 2e–h), and the other patients were considered negative due to symmetric distribution or calcifications (Figure 3). In all of these cases, [^68^Ga]FAPI-46 PET/CT accurately excluded metastasis in these lesions based solely on tracer uptake intensity.

**Fig. 4:**
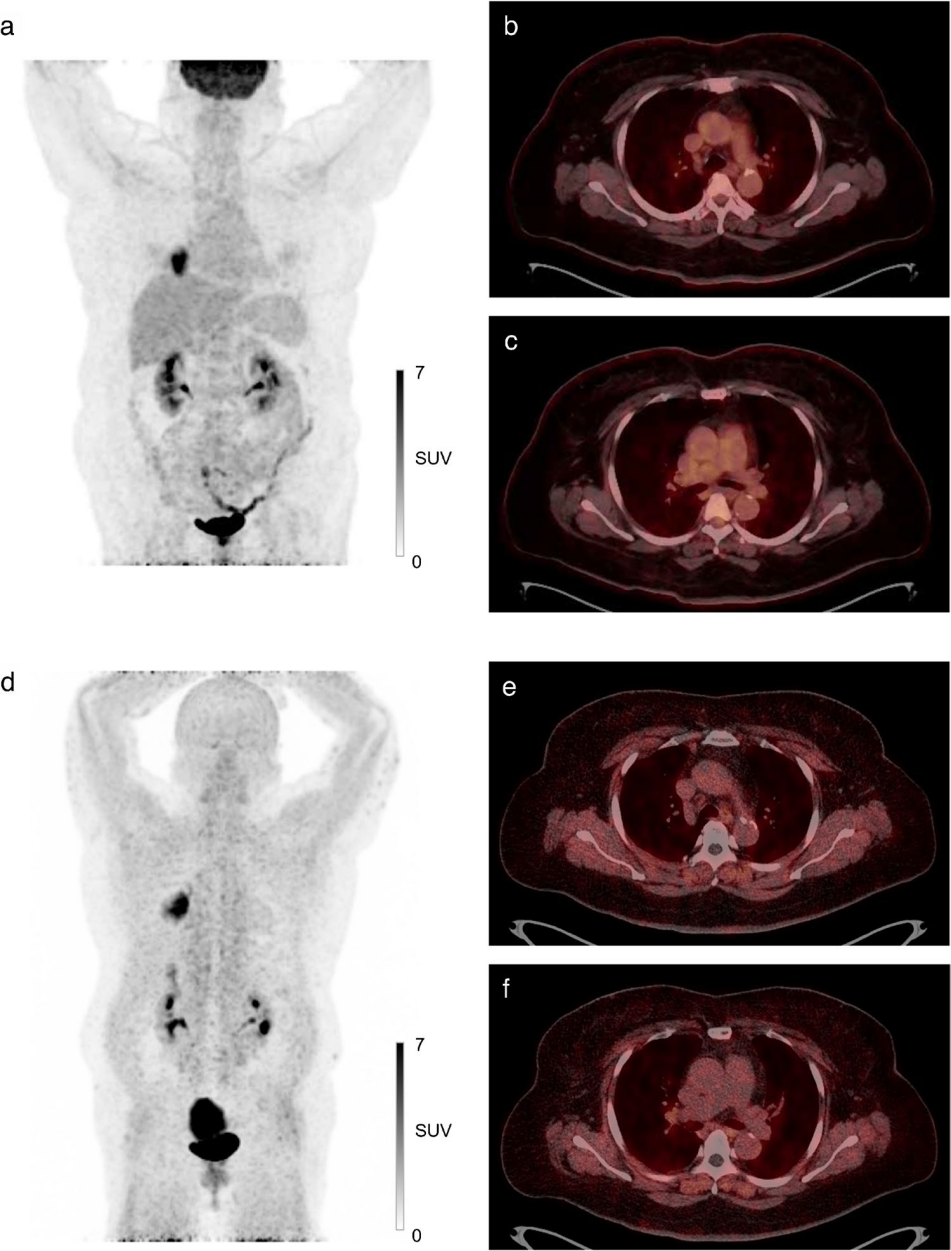
A case with false-negative findings on [^68^Ga]FAPI-46 PET/CT. A 62-year-old female patient diagnosed with lung adenocarcinoma. There was no lymph node with significant tracer uptake detected on [^68^Ga]FAPI-46 PET/CT (**a**–**c**) and [^18^F]FDG PET/CT (**d**–**f**). Metastasis was confirmed in stations 2R, 4R, and 7 in the postoperative histopathologic examination

The per-patient analysis resulted in one false-negative case in [^68^Ga]FAPI-46 PET/CT (Figure 4). In this case, only subcentimeter nodes without significant tracer uptake were observed on both scans, but postoperative histopathology revealed multiple N2 metastases. Increased tracer accumulation in the primary tumor suggests that insufficient tracer avidity was not the cause. The failure to detect these lesions might be attributed to inherent limitations of PET/CT in small-sized lesions resulting from low spatial resolution, rather than radiotracer characteristics.

A subgroup analysis was performed on nine patients who underwent EBUS-TBNA. Among them, five patients were diagnosed with N2 involvement by the pathologic results. [^68^Ga]FAPI-46 PET/CT accurately detected all five N2-positive patients, while EBUS-TBNA misdiagnosed one case to be non-metastatic (Table S1).

### Per-station assessment

**Fig. 5:**
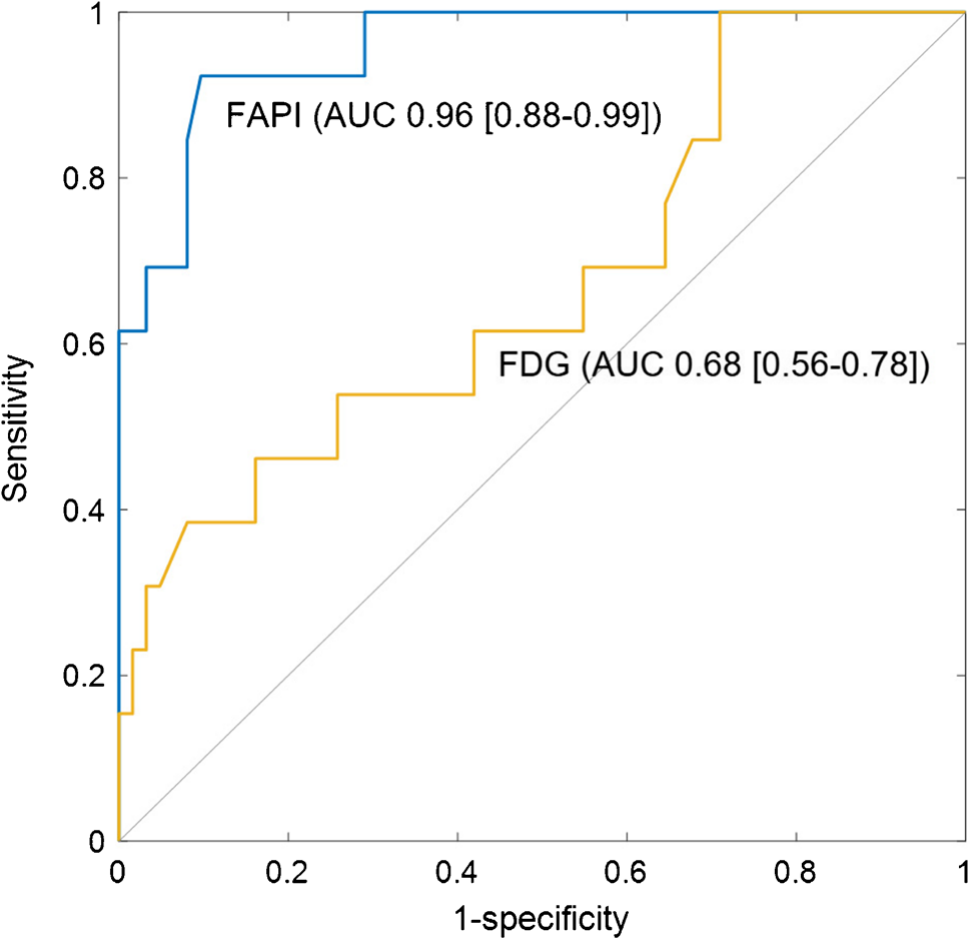
Per-station assessment of N2 mediastinal lymph nodes using [^68^Ga]FAPI-46 PET/CT and [^18^F]FDG PET/CT. Diagnostic performance of [^68^Ga]FAPI-46 PET/CT (*blue curve*) and [^18^F]FDG PET/CT (*yellow curve*) in per-station quantitative assessment of N2 mediastinal lymph nodes is represented by ROC curves. Values in the square brackets represent 95% confidence intervals. FAPI, fibroblast activation protein inhibitor; AUC, area under the curve

**Fig. 6:**
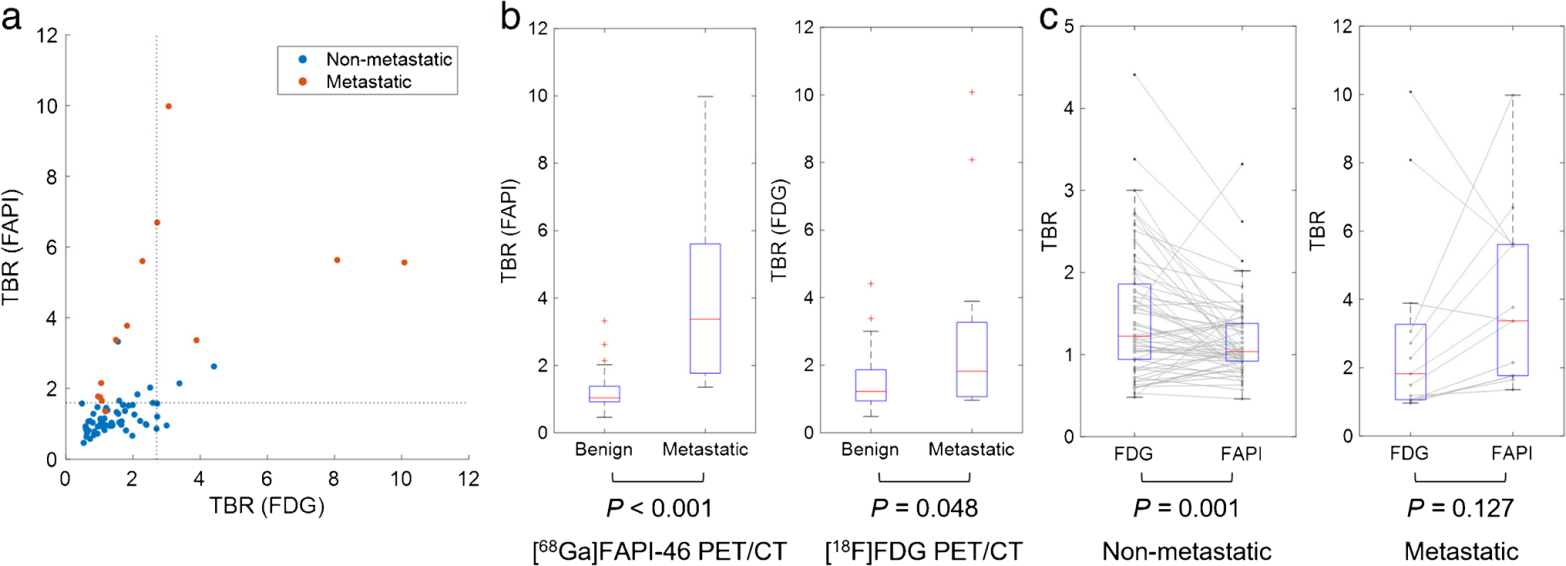
Comparison of tracer uptake in FAPI PET/CT and [^18^F]FDG PET/CT between non-metastatic and metastatic lymph nodes. **a** Comparison between tracer uptake in [^18^F]FDG PET/CT (*x* axis) and [^68^Ga]FAPI-46 PET/CT (*y* axis) in metastatic (*red*) and non-metastatic (*blue*) stations. Dot lines represent the optimal cutoff values to detect metastatic lesions. FAPI uptake discriminates metastatic nodal stations more effectively than FDG uptake. As detailed in Table 2, for true-positive N2 lesions, 12 out of 13 were identified above the threshold level with FAPI uptake, in contrast to only 5 with FDG uptake. The diagnostic values for true-negative lesions were not significantly different (6/62 vs. 5/62). **b** FAPI uptake and FDG uptake were compared between non-metastatic and metastatic stations with box-and-whisker plots, respectively. The middle lines indicate median values, and ends of the boxes represent the 25th and 75th percentiles. The whiskers extend to points that are 1.5 times the interquartile range from the box ends. **c** FAPI uptake and FDG uptake were compared in non-metastatic and metastatic stations, respectively. FAPI uptake was significantly lower in non-metastatic stations compared to FDG uptake. Conversely, FAPI uptake tended to be higher than FDG uptake, though this difference did not reach statistical significance. FAPI, fibroblast activation protein inhibitor; TBR, tumor-to-background ratio

A total of 75 N2 stations from 23 patients were surgically resected. Histopathologic examinations confirmed the presence of metastasis in 13 stations. In ROC curve analysis, the TBR of each PET/CT scan was used as a quantitative measure to differentiate metastasis. The TBR of [^68^Ga]FAPI-46 PET/CT exhibited favorable diagnostic performance in distinguishing metastatic nodes (AUC 0.96 (95% CI 0.88–0.99)) (Figure 5, blue curve), while TBR of [^18^F]FDG PET/CT demonstrated moderate performance (AUC 0.68 (0.56–0.78)) (Figure 5, yellow curve). The AUC of the ROC curve was significantly higher for TBR from [^68^Ga]FAPI-46 PET/CT compared to [^18^F]FDG PET/CT (*P* < 0.001). By employing the optimal cutoff values for TBR, [^68^Ga]FAPI-46 PET/CT (cutoff 1.59) correctly identified 12 out of 13 metastatic stations, while [^18^F]FDG PET/CT (cutoff 2.70) identified only five. With these cutoff values, the sensitivity and specificity for detecting metastatic stations were 0.92 (0.64–1.00) and 0.90 (0.80–0.96) for [^68^Ga]FAPI-46 PET/CT and 0.39 (0.14–0.68) and 0.91 (0.82–0.97) for [^18^F]FDG PET/CT (Table 2, and Figure 6a).

In the subgroup analysis of lesions examined by EBUS-TBNA, a total of fifteen nodal stations were included. Among them, pathologic analysis confirmed five metastatic stations, and four of them were detected by EBUS-TBNA accurately. [^68^Ga]FAPI-46 PET/CT identified all five metastatic stations correctly but misdiagnosed one non-metastatic station to be metastatic (Table S2).

### FAPI and FDG uptake of benign and metastatic lymph nodes

FAPI and FDG uptake in benign and metastatic nodal stations were compared using the TBR. Tracer uptake was higher in metastatic stations, both on [^68^Ga]FAPI-46 PET/CT (4.0 ± 2.5 vs. 1.2 ± 0.5, *P* < 0.001) and [^18^F]FDG PET/CT (3.0 ± 2.9 vs. 1.5 ± 0.8, *P* = 0.048) (Figure 6b). The difference in tracer uptake between benign and metastatic stations was more pronounced on [^68^Ga]FAPI-46 PET/CT, with a narrower border zone.

When comparing between FAPI and FDG uptake of each station, FAPI uptake was significantly lower than FDG uptake in non-metastatic stations (1.2 ± 0.5 vs. 1.5 ± 0.8, *P* = 0.001), whereas ten out of thirteen metastatic stations exhibited higher FAPI uptake than FDG, although the difference did not reach statistical significance (4.0 ± 2.5 vs. 3.0 ± 2.9, *P* = 0.127) (Figure 6c).

## Discussion

In this study, [^68^Ga]FAPI-46 PET/CT demonstrated promising results in detecting mediastinal nodal metastasis in a specifically selected group of NSCLC patients, where [^18^F]FDG PET/CT may exhibit limitations, potentially requiring preoperative EBUS-TBNA. [^68^Ga]FAPI-46 PET/CT accurately detected eight out of nine N2-metastatic patients while excluding all non-metastatic patients. It exhibited superior discriminative accuracy in the per-station assessment, resulting from a distinct difference in tracer uptake between non-metastatic and metastatic lesions.

Incorporating [^68^Ga]FAPI-46 PET/CT into clinical practice may offer dual benefits. First, its enhanced sensitivity upstaged 13.0% of patients without compromising specificity, reducing the risk of occult N2 metastases and guiding alternative treatments for high-stage patients who may not benefit from upfront surgery. Second, it distinguished metastatic stations based solely on tracer avidity. FDG is highly accumulated in granulomatous lesions, which necessitates the consideration of calcification and distribution patterns to enhance specificity. That leads to the compensatory expense of sensitivity [7, 8], and EBUS-TBNA is often performed even when observed nodes are considered as inflammation. In this context, [^68^Ga]FAPI-46 PET/CT has the potential to help avoiding invasive procedures.

Our results suggest the potential of [^68^Ga]FAPI-46 PET/CT to complement the standard staging process in resectable NSCLC. It is important to emphasize that our study was not designed as a head-to-head comparison with [^18^F]FDG PET/CT for the general NSCLC population. Instead, we specifically enrolled patients potentially indicated for invasive staging based on the established practice guidelines [2, 28], with the presence of FDG-positive N2 nodes as one of the inclusion criteria. That could impact the straightforward comparison of diagnostic performance between the two tests, and enrolled patients do not represent the entire NSCLC population. Our focus was rather on the specific situation where [^18^F]FDG PET/CT might have limited diagnostic value and invasive staging should precede the surgery. Our aim was to investigate whether [^68^Ga]FAPI-46 PET/CT can provide additive value in this situation, thereby correctly guiding the indication for EBUS-TBNA.

The use of FAPI PET/CT for mediastinal nodal staging has been explored in several studies. Wang et al. reported that [^68^Ga]FAPI PET/CT detected a higher number of metastatic nodes in advanced lung cancer than [^18^F]FDG PET/CT [24]. This study focused on the advanced stage, including recurrence state, and pathologic reference was not used. Zhou et al., in a post hoc retrospective subgroup analysis of 35 patients, reported accuracies of [^68^Ga]FAPI-04 and [^18^F]FDG PET/CT as 0.94 and 0.30, respectively [26], but this analysis only included FDG-avid lesions, producing the exceptionally underestimated accuracy of [^18^F]FDG PET/CT. Wu et al. reported the superiority of [^68^Ga]FAPI PET/CT in mediastinal nodal staging in a prospective study with 28 patients [25], demonstrating a sensitivity of 0.818 and specificity of 0.976, but MLND was performed in only ten patients. In contrast, our study specifically focused on its use for preoperative mediastinal nodal staging in operable NSCLC, emphasizing its clinical contribution particularly in guiding management plans for patients with potentially resectable NSCLC and ensuring all lesions are evaluated with postoperative pathologic confirmation.

One of our key objectives was to assess the role of [^68^Ga]FAPI-46 PET/CT in effectively stratifying patients who may require invasive staging. This goal was indirectly addressed by demonstrating its superior accuracy over [^18^F]FDG PET/CT and its potential to refine the indication for proceeding to invasive procedures. It is important to note that EBUS-TBNA was not a routine part of our protocol, which precluded a direct comparison in the main analysis. However, we conducted a subgroup analysis on a limited cohort (9 patients with 15 stations) who underwent EBUS-TBNA. The results were somewhat promising, showing a per-station accuracy of 0.93 for both [^68^Ga]FAPI-46 PET/CT and EBUS-TBNA in detecting N2 metastasis. In addition, the overall sensitivities of [^68^Ga]FAPI-46 PET/CT in the entire population appear to be not significantly inferior to that of EBUS-TBNA reported in a previous study (a pooled sensitivity of 0.89) [28]. Despite the limited cohort size, these findings may indicate the potential of [^68^Ga]FAPI-46 PET/CT to refine the existing mediastinal staging process that includes [^18^F]FDG PET/CT and EBUS-TBNA, warranting the need for further prospective studies to compare its diagnostic accuracy with these conventional tests in a larger population.

We focused on “N2” metastasis in the analyses. The accurate staging of mediastinal nodes is pivotal in determining optimal treatment strategies for patients with resectable NSCLC [1, 2]. In particular, N2 involvement plays a critical role in the decision-making process for curative surgery or adjuvant therapy. Furthermore, focusing specifically on N2 stations enables reliable comparison between PET/CT and postoperative histopathology, because MLND generally covers only N1 and N2 regions and reliable correlation with tomographic locations is limited in N1. For these reasons, we decided to analyze only N2 regions.

The relatively longer intervals between [^68^Ga]FAPI-46 PET/CT and [^18^F]FDG PET/CT could potentially impact the comparative results of the two tests. However, it is important to note that our study did not include patients with highly advanced disease stages or poorly differentiated or small cell pathology. This factor reduces the likelihood of rapid tumor progression within this interval. To further address this concern, we performed a supplementary analysis comparing the diameters of metastatic nodes as measured on [^68^Ga]FAPI-46 PET/CT and [^18^F]FDG PET/CT (Figure S1). The results showed no significant differences in the diameters measured on both scans, suggesting that any tumor progression during the intervals was not substantial enough to impact the results.

Although CECT is a standard imaging modality in lung cancer staging, it was not part of the analysis in our study. CECT is the most crucial imaging tool in lung cancer, offering the most accurate clinical T stage determination, screening for intrathoracic metastatic lesions, and providing essential anatomical information needed for histologic sampling and surgical approaches. However, the diagnostic efficacy for mediastinal metastasis is acknowledged to be limited, as supported by established practice guidelines [28]. The guideline reported, through meta-analyses, pooled sensitivity and specificity of CECT in mediastinal staging to be 0.55 and 0.81, which were less than those of [^18^F]FDG PET/CT (0.62 and 0.90). In our study, we excluded CECT from the analyses to focus on the role of [^68^Ga]FAPI-46 PET/CT as a method to address the limitations of [^18^F]FDG PET/CT.

This study has limitations to be addressed. As a pilot study, we collected the data from a limited number of patients. As described above, many patients did not undergo EBUS-TBNA. We indirectly showed the feasibility of [^68^Ga]FAPI-46 PET/CT to reduce the need for EBUS-TBNA by exhibiting its high diagnostic accuracy, but the estimation for this potential is limited due to the lack of sufficient head-to-head comparison. Additionally, mediastinal lymph nodes were resected selectively based on surgeons’ intraoperative assessments as in routine clinical practice, which could influence the precise evaluation of the sensitivity and the false-negative rate. The predominance of patients with adenocarcinoma may also limit the generalizability of the findings and their interpretation across the different pathologic subtypes. The results need to be validated with a larger prospective study designed to encompass broader disease stages and enable the direct comparison among different diagnostic methods.

## Conclusion

We report the results of the pilot study on [^68^Ga]FAPI-46 PET/CT in mediastinal nodal staging of NSCLC. [^68^Ga]FAPI-46 PET/CT offered higher diagnostic accuracy in distinguishing metastatic N2 lymph nodes in patients who may be indicated for invasive staging. This result suggests the feasibility of accurate selection of patients who require invasive staging procedures by incorporating [^68^Ga]FAPI-46 PET/CT into practice. A larger prospective study is necessary to establish its role.

### Supplementary information

Below is the link to the electronic supplementary material.

Supplementary file1 (DOCX 56.3 KB)

## Data Availability

The datasets generated during and/or analyzed during the current study are available from the corresponding author on reasonable request.
