# Supplementary material for: Preoperative evaluation of mediastinal lymph nodes in non-small cell lung cancer using [68Ga]FAPI-46 PET/CT: a prospective pilot study
Source: Eur J Nucl Med Mol Imaging. 2024 Mar 7;51(8):2409–19. doi: 10.1007/s00259-024-06669-y (PMC11178623; doi:10.1007/s00259-024-06669-y)
Supplement: Supplementary file 1 — Supplementary file1 (DOCX 56.3 KB) [file 259_2024_6669_MOESM1_ESM.docx]

**Supplementary Data**

**Preoperative evaluation of mediastinal lymph nodes in non-small cell lung cancer using [^68^Ga]FAPI-46 PET/CT: A prospective pilot study**

Yeon-koo Kang, MD, PhD ^a,d†^, Kwon Joong Na, MD ^b,e†^, Jimyung Park, MD^c^, Nakwon Kwak, MD, PhD^c^, Yun-Sang Lee, PhD ^a,d,f^, Hongyoon Choi, MD, PhD^a,d,f,*^ and Young Tae Kim, MD, PhD ^b,e*^

^a^Department of Nuclear Medicine, Seoul National University Hospital, Seoul, Republic of Korea;

^b^Department of Thoracic and Cardiovascular Surgery, Seoul National University Hospital, Seoul, Republic of Korea;

^c^Division of Pulmonary and Critical Care Medicine, Department of Internal Medicine, Seoul National University Hospital, Seoul National University College of Medicine, Seoul, Republic of Korea;

^d^Department of Nuclear Medicine, Seoul National University College of Medicine, Seoul, Republic of Korea

^e^Cancer Research Institute, Seoul National University College of Medicine, Seoul, Republic of Korea

^f^Institute of Radiation Medicine, Seoul National University Medical Research Center, Seoul, Republic of Korea

^†^These authors contributed equally.

***Corresponding Author:**

**Hongyoon Choi, MD, PhD**

Department of Nuclear Medicine, Seoul National University Hospital

101 Daehak-ro, Jongno-gu, Seoul 03080, Korea

Tel: +82-2-2072-3341, Fax: +82-2-745-7690, E-mail: chy1000@snu.ac.kr

**Young Tae Kim, MD, PhD**

Department of Thoracic and Cardiovascular Surgery, Seoul National University Hospital

101 Daehak-ro, Jongno-gu, Seoul 03080, Korea

Tel: +82-2-2072-3161, Fax: +82-2-764-3664, E-mail: ytkim@snu.ac.kr

**List of supplementary data**

**Table S1.** Per-patient diagnostic accuracy for detecting N2 metastasis in patients who underwent EBUS-TBNA.

**Table S2.** Per-station diagnostic accuracy for detecting N2 metastasis in patients who underwent EBUS-TBNA.

**Figure S1.** Comparison of metastatic node sizes on [^18^F]FDG PET/CT and [^68^Ga]FAPI-46 PET/CT

**Table S1.** Per-patient diagnostic accuracy for detecting N2 metastasis in patients who underwent EBUS-TBNA.

| **Per-patient analysis (visual assessment for FAPI PET/CT)** | | | | | |
| --- | --- | --- | --- | --- | --- |
|  | FAPI-positive | FAPI-negative | EBUS-positive | EBUS-negative | All |
| N2-positive | 5 (100.0%) | 0 (0.0%) | 4 (80.0%) | 1 (20.0%) | 5 |
| N2-negative | 0 (0.0%) | 4 (100.0%) | 0 (0.0%) | 4 (100.0%) | 4 |
| All | 5 | 4 | 4 | 5 | 9 |

**Table S2.** Per-station diagnostic accuracy for detecting N2 metastasis in patients who underwent EBUS-TBNA.

| **Per-station analysis (with determined optimal cutoff for FAPI uptake)** | | | | | |
| --- | --- | --- | --- | --- | --- |
|  | FAPI-positive | FAPI-negative | EBUS-positive | EBUS-negative | All |
| N2-positive | 5 (100.0%) | 0 (0.0%) | 4 (80.0%) | 1 (20.0%) | 5 |
| N2-negative | 1 (10.0%) | 9 (90.0%) | 0 (0.0%) | 10 (100.0%) | 10 |
| All | 6 | 9 | 4 | 11 | 15 |


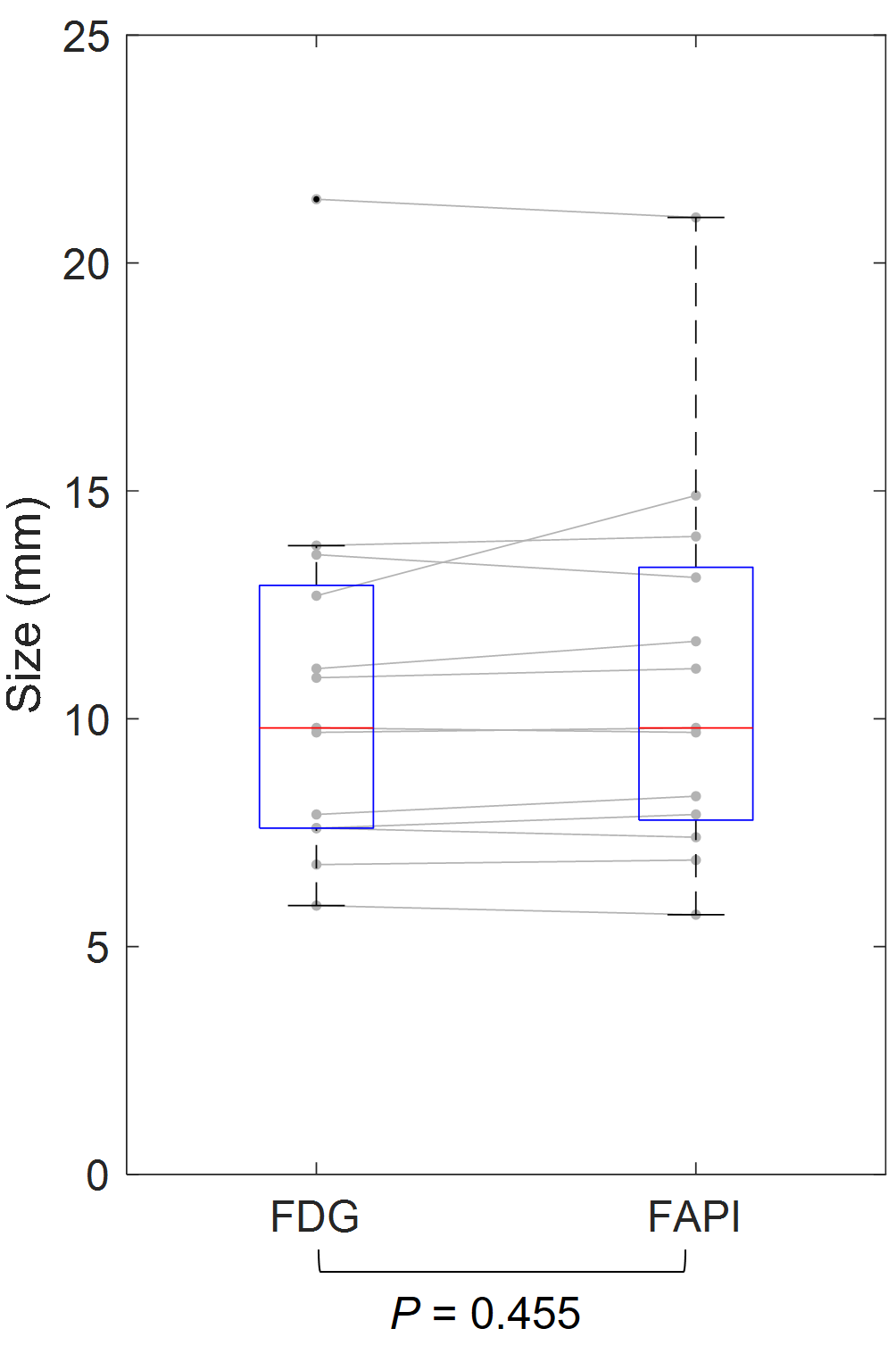


**Figure S1.** Comparison of metastatic node sizes on [^18^F]FDG PET/CT and [^68^Ga]FAPI-46 PET/CT

The largest diameters of all metastatic N2 nodes as measured on [^18^F]FDG PET/CT and [^68^Ga]FAPI-46 PET/CT were compared using the Wilcoxon signed-rank test. There was no significant difference in node sizes between the two test, indicating that the time intervals between the scans did not impact the results. The only lesion that exhibited size an increase in size during the interval (from 12.7 mm to 14.9 mm) was identified as metastatic on both scan, not affecting the comparative outcomes. The middle lines indicate median values, and ends of the boxes represent the 25th and 75th percentiles. The whiskers extend to points that are 1.5 times the interquartile range from the box ends.
